# Community health learning experiences of Colombian undergraduate medical students. A phenomenographic research study

**DOI:** 10.1007/s10459-024-10395-3

**Published:** 2025-02-14

**Authors:** Claudia Liliana Jaimes-Peñuela, Francisco Lamus-Lemus, Natalia Reinoso-Chávez

**Affiliations:** 1 Department of Family Medicine and Public Health, School of Medicine, Universidad de La Sabana, Chía, Cundinamarca, Colombia; 2 Independent Researcher and Scholar, Chía, Cundinamarca, Colombia

**Keywords:** Medical education, Undergraduate, Learning experience, Transformative service learning, Community health education, Phenomenography

## Abstract

Medical students’ perceptions of their community learning experiences can provide valuable insights for evolving and improving healthcare professional education curricula to better respond to health needs. This study aims to explore the learning experiences of undergraduate medical students in a community health course at a Colombian University. Fifteen students who had completed the Family Medicine and Community Health course participated in this qualitative study, employing a phenomenographic approach and interview technique. The phenomenographic analysis, informed by the transformative service-learning theoretical framework, resulted in the construction of categories of description, organized hierarchically, based on levels of understanding. The findings revealed five main community health learning experiences: Fulfilling the requirements, Educating the community, Solving health problems, Joint construction, and Personal transformation. Students experienced learning in diverse hierarchical learning levels, with their understanding around community health evolving over time. Transformative learning experiences were identified among only some students, indicating the potential for everyone to reach such levels of complexity and depth. Educationally critical aspects for achieving more complex levels of learning include exposure to intense and major challenges in longer periods of community services, reflection guided by teacher in intentional pedagogical spaces, feedback, and meaningful relationships between key stakeholders. These findings hold significance for medical programs that offer community medicine or related courses, since they present pedagogical opportunities to create and improve similar learning contexts in other domains.

**Supplementary Information:**

The online version contains supplementary material available at 10.1007/s10459-024-10395-3.

## Introduction

In Colombia, healthcare faces challenges related to fragmentation and segmentation of service delivery, resulting in concentrated health expenditure on supra-specialized services that inadequately address community-level health determinants (Hernández-Rincón, 2017). Challenges related to the provision of quality care and general levels of health equity are increasingly common throughout Latin America (Ruano et al., 2021) and in several countries around the world even though their magnitude and primary causes may differ (Gebremeskel et al., 2023). While physical, financial, and technological resources are undoubtedly necessary for providing adequate healthcare, human health resources are essential for ensuring comprehensive approaches to improving health conditions and satisfaction with healthcare at the community level (Hernández Morales et al., 2018).

Traditionally, medical education has primarily focused on disease identification and treatment within a hospital and curative paradigm (Greer et al., 2018). However, health professional training must evolve alongside current societal changes, reorienting itself toward Primary Health Care focused on the patients’ and the communities’ evolving needs and circumstances (Arias-Castillo, 2019; Frenk et al., 2010). In this study, we explore the learning experiences of undergraduate medical students in a community health course that addresses this purpose at a Colombian University.

Organizations around the world encourage partnerships between academic leaders and community members to address local health concerns (Holden et al., 2016; Hunt et al., 2011; Ministerio, 2017), and service-learning strategies contributes to this endeavor (Playford et al., 2019). Service-learning is a set of educational practices where community work and formal education converge (Lalueza et al., 2016), rooted in experiential learning (Schank & Halberstadt, 2023). It emphasizes active community participation and ongoing reflection, sending learners to serve in a community that is different from their own, and then bringing them back to reflect on their experiences lived through in a learning environment (Playford et al., 2019; Schank & Halberstadt, 2023).

One of the key benefits of service learning is its potential for longitudinal integration (Arebalos et al., 2021). For undergraduate medical students who engage in learning and teaching experiences that embody this longitudinal aspect, the benefits are substantial (Ellaway et al., 2013). For example, students develop adaptive strategies to manage the complexities of clinical practice, leading to significant growth in their confidence and competence as future physicians (Dubé et al., 2015). Additionally, there have been notable improvements in students’ perceptions of assessment and feedback, which are now viewed as more authentic, useful, and constructive due to their integration into daily patient care and the supportive learning environment (Bates et al., 2013). Furthermore, students’ attitudes toward underserved populations remain stable when they interact with these communities at their service-learning sites, rather than deteriorating as previous studies have often shown (Arebalos et al., 2021). Although not all service-learning experiences include a longitudinal component, its presence offers significant advantages.

In Colombia, documented service-learning experiences in undergraduate medical programs have primarily been explored from the perspectives of faculty and community leaders (Lamus-Lemus et al., 2017). However, while students’ learning experiences in community health have been documented, studies focusing on their perspectives are relatively few within the Colombian context (Pimentel et al., 2020, 2022; Schneider et al., 2018). Understanding students’ experiences is crucial, as their insights can drive improvements in healthcare professional education, especially in resource-limited settings.

## Theoretical framework

### Transformative service-learning for community medicine competencies

Transformative Service-Learning (TSL) is a pedagogical model in which students are immersed in a community, involving individual and social transformation processes derived from this experience (Naudé, 2015). This approach has been widely used to develop key competencies in healthcare professionals, facilitating the transformation of their knowledge, perspectives, and attitudes. It helps them become more socially aware, responsible, and patient-oriented practitioners who are respectful, compassionate, and empathetic toward the struggles and strengths of their patients and peers (Johnson & Howell, 2017; Ng et al., 2021; Yang et al., 2021). In disciplines such as community psychology, proving to be particularly useful to develop inter and intrapersonal skills for community interventions that require experiential learning (Trigos-Carrillo et al., 2020).

Kiely (2005) TSL Model draws from Mezirow’s (1991) transformational learning theory and other empirical studies. In Kiely’s model, there are five stages outlining students’ transformational learning in service-learning: (1) contextual border crossing which describes four elements of context (personal, structural, historical, and programmatic factors) that influence students’ transformational learning; (2) dissonance, suggesting that students’ experiences in service-learning can be incongruent with their current worldview; (3) personalizing, where students begin responding in an emotional way to the different forms of dissonance; (4) processing with students, cognitively processing their service-learning interactions and experiences and ultimately, (5) connecting, leading to connections with community individuals and their problems (Kiely, 2005; Whitley, 2014).

Steps toward transformational learning require diverse learning conditions to be successful, creating opportunities for innovative teaching and learning arrangements. While existing documentation on TSL experiences in health professions education often focuses on elective courses with short durations (Johnson & Howell, 2017; Ng et al., 2021; Trigos-Carrillo et al., 2020; Yang et al., 2021), it is essential to explore TSL experiences from the perspective of students in mandatory undergraduate medical programs that involve longer durations and employ participatory project management practices in underserved community settings.

The study aims to comprehend the diversified learning experiences of undergraduate medical students in a community health course at a Colombian University, from their own perspective. The following objectives were raised:

1. Explore the learning experiences of undergraduate medical students in a community health course.
2. Identify pedagogical opportunities for enhancing healthcare professional education curricula within community settings.

Therefore, we will present learning experiences at different levels of complexity, identifying critical aspects that can improve learning towards a transformational process. Results of this study contribute to our understanding of what it takes to learn and how health professional students learn community interventions skills in service-learning scenarios. Teachers could use this information when teaching (Holmqvist & Selin, 2019) and therefore, invaluable for pedagogical purposes (Kettunen & Tynjälä, 2022).

## Methods

### Study context

The study was developed in the context of a Medicine undergraduate course offered by a private university in Colombia since 1999 to teach community medicine key competences. The course is developed in partnership with institutions attending vulnerable communities (rural elders, poor women or children, farm workers of rural nearby settings). The study involved 15 students, organized into groups of four to six, who selected a community to work on a community health project during the 11th semester of a 7-year (14-semester) undergraduate medical program. Following the course design, these groups joined an established initiative between the community and the University, forming community-campus alliances that aimed to promote healthier community environments. Following the service-learning approach, each group worked on their project for an 18-week period, under the guidance of a tutor (academic university staff). They identified community health needs and were involved in the design, implementation, and evaluation of a community health plan, actively engaging community members and other local stakeholders. This process, known as the community health cycle, integrates elements from the iterative process of the Kansas University Community Toolbox (Center for Community Health & Development, 2022), the planning paradigm model of change theory, teachability, governance, and cultural adaptation principles integrated by the community medicine faculty at Universidad de La Sabana (Lamus-Lemus et al., 2017).

### Study design

A qualitative study using a phenomenographic approach was conducted with students from five different cohorts who took the course between the second semester of 2017 and the second semester of 2019.

Phenomenography is a research method designed to map the diverse ways individuals experience, conceptualize, perceive, and understand phenomena in their world (Marton, 1986). It is situated within the interpretative paradigm, which focuses on understanding the subjective meanings and experiences of individuals (Åkerlind, 2005). Phenomenography is increasingly applied in educational and health research (Edström et al., 2015; Han & Ellis, 2019; Stenfors-Hayes et al., 2013; Wright & Osman, 2018).

The study examines both the referential aspect (the meaning of the phenomenon) and the structural aspect (specific elements of attention) (Röing et al., 2018). The concept of *dimensions of variation* is utilized to define and differentiate ways of experiencing and understanding learning (Marton, 2015; Marton & Booth, 1997; Marton & Tsui, 2004). This approach is essential for identifying the needs for a more complex learning experience (Åkerlind, 2015; Kettunen & Tynjälä, 2022; Wright & Osman, 2018) and enables educators to address these differences to enhance teaching and learning (Åkerlind, 2015; Orgill, 2012).

In phenomenography, outcomes are organized into *categories of description* and an *outcome space. Categories of description* capture the varied ways in which a phenomenon may be experienced, while the *outcome space* illustrates the structural relationships among these experiences (Åkerlind, 2005; Röing et al., 2018). For example, recent studies by Mimirinis and Wright (2024) and Zou et al. (2020) further demonstrate how an outcome space can elucidate structural connections underlying varied experiential perspectives—in this case, academic achievement among Black students and conceptions of teaching excellence among award-winning educators, respectively. These studies provide valuable additional support for the development of the outcome space in the present study, enhancing its ability to represent the complex relationships inherent in Colombian medical students’ community health learning experiences. This nuanced understanding is essential for shaping educational practices that resonate with students’ varied understandings.

### Recruitment

The population for this study was purposefully selected to ensure maximum variation (González-Ugalde, 2014) following three principles: (a) subjects with experience of the phenomenon under study (Marton & Booth, 1997); (b) men and women of different ages, who took the community health course in different years and communities, thus maximizing the variation (Bowden, 2000) and (c) the participation of a suitable number of subjects would allow finding existing variations until no further variability is found (Dunkin, 2000; Trigwell, 2000).

We compiled a list of students who completed the immersion semester in Family Medicine and Community Health between the second semester of 2017 and the second semester of 2019. In accordance with Bowden’s (2000) recommendation, we aimed to capture variation in experience by considering factors such as gender, age, community type, tutor (academic university staff), and academic cohort. Invitations were sent to all individuals on the list (43 in total), of whom 15 expressed interest and agreed to participate. These students were subsequently contacted via personal text messages to schedule interviews and to present the informed consent form. Refer to Table 1 for detailed information.

**Table 1:** Participant characteristics

Reference*	Sex^a^	Age (years)	Course year	Community
Diego	M	29	2017 Jul-Nov	Kindergarten (children under 6 years old)
Diana	F	24	2019 Jul-Nov	
Marina	F	25	2017 Jul-Nov	Caregivers of older adults
Melissa	F	27	2017 Jul-Nov	NGO^b^ assisting children (0–5 years) and mothers
Carolina	F	23	2019 Jan-May	
Silvia	F	23	2018 Jul-Nov	Religious community of older women
Juanita	F	24	2019 Jan-May	NGO attending children and adolescents under state protection
Alexandra	F	26	2019 Jul-Nov	
Paola	F	24	2019 Jul-Nov	
Andrés	M	23	2019 Jan-May	University community
Mónica	F	25	2018 Jul-Nov	Outpatient center that provides services to street dwellers
Lina	F	24	2018 Jul-Nov	
Camilo	M	22	2019 Jul-Nov	Teenagers from a public school
Javier	M	23	2019 Jul-Nov	
Carlos	M	25	2018 Jul-Nov	Pregnant women and their families

* Names given are pseudonyms.

^a^ M = male; F = female.

^b^ non-governmental organization.

To guarantee the anonymity and confidentiality of the information, no data that could be linked to the participants was mentioned and pseudonyms were used.

### Instrument: phenomenographic interview

Phenomenographic interviews are characterized by, firstly, its intention is to find variation in participants’ perspective on the experience of a phenomenon, which may vary even among the same individual; and secondly, the interview centers on how the topic of the interview is experienced by the participants (Tan & Tan, 2020).

An interview protocol was developed based on literature review and expert opinions (appendix 1). Its purpose was to explore the various ways of experiencing learning and the relationships established with all its elements. A pilot test was conducted with three participants, whose feedback helped refine the interview guide and their responses were included in the study results.

### Data collection

The interviews were conducted between March and May 2020. Initially held in person at university facilities, they were subsequently moved to a remote format due to the COVID-19 pandemic.

All interviews were audio-recorded and transcribed verbatim by the interviewer (CJ), with an average duration of 38 min.

### Data analysis

The phenomenographic analysis followed the steps outlined by González (2010):

1. The first author, CJ, conducted multiple readings of the transcripts to familiarize themselves with the data and to identify sections pertinent to the research question.
2. CJ then performed a focused reading to identify similarities and differences in the relevant sections, documenting illustrative citations.
3. The initial analysis generated a set of *categories of description*, which were presented and discussed with the other researchers (FLL and NRC). The number of categories was subsequently reduced after determining that some described the same concept under different labels.
4. CJ re-read the transcripts in relation to the initial *categories of description*, with the aim of determining whether the categories accurately captured the learning experiences of the medical students. This iterative process involved continually testing and refining the categories until the system of meaning was stabilized.
5. CJ, FLL, and NRC collaboratively constructed the *outcome space*, organizing the *categories of description* hierarchically, where simpler categories were encompassed by more complex ones.

Lastly, a metaphor was assigned to each category of description to facilitate a more intuitive understanding of its underlying content.

### Trustworthiness

An audit trial was maintained by CJ who kept a detailed journal documenting emerging findings and methodological considerations throughout the research process. This practice ensured transparency and traceability, facilitating a clear understanding of how conclusions were reached (Collier-Reed et al., 2009). Extracts from the interviews were analyzed and categorized (Rands & Gansemer-Topf, 2016). This categorization provided context and ensured that interpretations were rooted in the participants’ perspectives (Collier-Reed et al., 2009).

Additionally, all authors have experience with the phenomenon of interest. Ongoing peer debriefing sessions were conducted with the second and the third author (Han & Ellis, 2019). These discussions critically examined the research process, findings, and interpretations, helping to identify and address potential biases and enhance the accuracy of the analysis (Collier-Reed et al., 2009).

Detailed reflexivity and positionality statements to acknowledge and address the researchers’ perspectives and potential influences on the study were also included. This self-awareness contributed to the integrity of the research by explicitly recognizing any biases or assumptions that could impact the findings (Collier-Reed et al., 2009).

## Findings

To present the diverse learning experiences in community medicine, we firstly present the *categories of description* (Table 2). Secondly, we present the *dimensions of variation* (Table 3), and thirdly, the *outcome space* (Table 4).

**Table 2:** Structural and referential aspects of the categories of description

Categories of description	Referential aspect	Structural aspect (focus)
A. Fulfilling the requirements—“Learner”	Following of the curriculum established by the University	Tasks and grade scores
B. Educating the community—“Teacher”	Provide health knowledge to the community	Community health knowledge
C. Solving health problems—“Hero”	Attention to community health problems and needs	Community health problems and needs
D. Joint construction—“Builder”	Participation in a team to build opportunities for improvement	Teamwork to solve real community problems
E. Personal transformation—“Pilot”	Recognition of contrasts in realities, personal growth, and humanization of the healthcare services	Contrasts of realities, personal and professional experiences

**Table 3:** Description of the dimensions of variation

Dimensions of variation	Challenges	Relationships	Learning strategies	Skills
Description	It refers to the challenges that the students faced during the community health course	It refers to the relationships that the students established with members of the community, academic peers, and university academic tutors, during the community health course	It refers to the various activities undertaken by students in their learning processes during educational interactions within community settings	It refers to the skills that the students developed during the community health course

**Table 4:** Outcome space: categories of description and dimensions of variation of community health learning experiences of Colombian undergraduate medical students

Categories of description	Dimensions of variation			
Categories	Challenges	Relationships	Learning strategies	Skills
A. Fulfilling the Requirements—"Learner"	Completing assigned tasks	Distant and respectful	Following instructions	Scientific writing and technical medical skills
B. Educating the Community—"Teacher"	As A, and contextualized teaching	Vertical-expert	As A, and consolidation of previous knowledge	As A, and health education and communication skills
C. Solving Health Problems—"Hero"	As B, and community work	As B	As B, and application in real scenarios	As B, and service orientation to solve problems
D. Joint Construction—“Builder”	As C, and teamwork	Horizontal (equality of status), and alliances	As C, and integration of personal and professional experiences; close relationships	As C, and (Leadership, partnership, teambuilding, and conflict management)
E. Personal Transformation—“Pilot”	As D, and transforming personal believes	As D, and affective ties	As D, and guided reflection and constant feedback	As D, and self-awareness comprehensiveness and humanization; empathy: understanding of other; supportive relationships personal and professional confidence

*Categories of description* represent the different ways in which students experienced community health learning, differentiating the intertwined referential and structural aspects portrayed in the emerging categories. The assigned metaphors represent the way students relate to learning and should not be understood as a typology of the role assumed. Illustrative statements are presented in each category.

Secondly, four *dimensions of variation* are presented to provide a richer view of community health learning experiences and to describe the relationships between these experiences in a service-learning context. These dimensions are illustrated with excerpts from interviews.

Finally, in the *outcome space*, the internal relationships of the *categories of description* are hierarchically organized, using the transformative service-learning theoretical framework as a guide.

### Categories of description

#### Category A: Fulfilling the requirements—"Learner"

For some of the students, learning was experienced as the fulfillment of an academic requirement—following the Medical undergraduate curriculum established by the University, where community health is compulsory and not an elective course. The focus was on assigned tasks, care activities and deliverables, and the grade scores they generated. Students’ actions aimed to achieve grades that allowed them to pass the course and advance in their professional training.

"We must do a good job. At the end of the day, the reason why we do something at that moment, is to pass the exams."—Juanita

#### Category B: Educating the community—"Teacher"

Another type of experience understood community health learning mainly as an act of education towards others—providing the knowledge that the community needed. The focus was on the knowledge that students thought community members lacked. Therefore, student`s actions were initially oriented to community knowledge evaluation and later, to facilitate knowledge acquisition, to prevent diseases and maintain health.

"If the child had the flu, I taught them what to do: nasal rinsing, hands washing, mask-wearing. I was always teaching them what I knew."—Melissa

#### Category C: Solving health problems—"Hero"

In another range of experience, learning was centered in attending to health needs of the community. The focus was precisely on those unmet needs. Thus, the student’s actions were initially oriented to lead the search and prioritization of relevant health issues and later, to the planning and execution of strategies to solve those problems.

"Community health practitioner assesses a health problem in a community, to prevent and solve problems of this population."—Camilo

#### Category D: Joint construction—"Builder"

Learning was experienced as participating in a team to build community improvement opportunities. The focus was on teamwork in real situations and on the achievements, that is, on the products generated as a team. Therefore, the student’s actions always involved the participation of other individuals: peers and community.

"What you do in a hospital is to follow instructions. What you do in the community is to build something. We all build something to make it work—not for me, not for my knowledge, but for and with the community."—Silvia

#### Category E: Personal transformation—"Pilot"

For some students, learning was experienced as the recognition of other sociocultural realities, which led the student to develop new personal and professional perspectives identified as opportunities for personal growth and humanization of their profession.

"At that time, I learned to value many things that I did not value before. They know the value of a hug, a call from a family member or a friend, quite simple things, that one may always have, but does not value. For me, learning that was great."—Lina

The focus extended to different political, social, and economic realities, personal experiences, and professional life in the short and long term, including postgraduate studies and work practice.

"The fact of becoming more human and remembering that you treat a patient who, at that moment due to his illness or his problem, is a vulnerable person, and who has to be respected and understood. The problem with us as doctors is that, over time we forget about it, and this course reminded me of it again."—Alexandra

## Dimensions of variation

This study identified four *dimensions of variation* of learning community medicine in service learning-experiences, which were based on aspects and their features identified in the data: challenges faced, relationships with community, peers, and tutor, learning strategies and skills developed. These *dimensions of variation* broadened the vision guiding us to understanding educationally critical aspects involved in more complex and transformative learning and allowed the categories to be related to each other.

### Challenges

In category A, challenges were related to the assigned academic tasks assigned, such as literature search, data tabulation, and writing scientific articles. In category B, in addition, challenges were related to the teaching role assumed by the students, including healthcare education strategies and educational tools.

"The biggest challenge was when we had to tabulate all the data, when we had to make the conclusions, try to be as objective as possible."—Lina

"Sometimes you shut yourself up in medicine, in your world, and you think that people are going to understand your technicalities. And it’s not like that."—Silvia

In categories C and D, the challenges involved community work, such as commuting, schedules, communication skills, and creating and maintaining trust. In category D, there were also challenges with teamwork: reaching agreements, sharing leadership, and putting the healthcare professional’s ego aside.

"Getting the schedules to match, both: ours and theirs, was a bit complicated, but beyond that, it was not to lose contact."—Carlos

"In the working group there were two leaders. At first, we argued because we both wanted to lead."—Melissa

In addition to the above, in category E, challenges were related to personal aspects, including fears (rejection and insecurity), pre-established judgments, and facing the characteristics of vulnerability of the community: poverty, violence, and violation of human rights.

"We realized that in most places there was no stove or gas, but little kitchens; next to the bed, next to the bathroom, there was no door but curtains, there were no walls but cloths."—Melissa

### Relationships

In category A, the relationships were based on mutual respect and cooperation, with the sole purpose of fulfilling academic assignments. The student saw him or herself as an individual and was not part of a group.

"Hopefully it will be people who work so that one is not going to play so hard and can have good grades, because it is what one looks for at the end...And that really happened at the beginning, well we don’t know each other, but being able to get this out the best you can, the best grades."—Juanita

In categories B, C and D the relationships were collaborative. In category B, the student saw him or herself as an individual and sometimes identified him or herself as part of the group. In category C, the student saw him or herself as an individual who was part of the group. Whereas, in category D, the student did not conceive him or herself outside the team.

"At the end of the day, we all did our part and, everyone recognized what they liked."—Diana

"Do not forget that we as doctors work as a team, regardless of whether we have knowledge or not, medicine will always be teamwork. It is a whole, if one of the parts fails, possibly the care that one hopes to provide will not be provided."—Carlos

Furthermore, in category D, interactions characterized by equality of status among participants highlighted a distinct teacher-student relationship compared to other rotations. Teachers were viewed as "tutors" who were integrated into the team, fostering a safe space for both learning and working.

"Not only did we see the doctor as the tutor, our teacher, but she was also another colleague, another leader, who not only helped us with ideas, but also felt that family atmosphere. We felt she was part of the group."—Carlos

In category E, emotional bonds were also established with all those involved (community members, academic peers, and tutors) facilitating teamwork and generating a commitment.

"We liked and were comfortable with each other. There was a beautiful relationship between us, so we did not see it as an obligation...That happened because it was not a relationship neither of doctor-community, nor of medical student-community, but of people sharing experiences as equals."—Melissa

### Learning strategies

In category A, students followed instructions from tutors and the community.

"We develop the project as such, by means of a written report, in which we begin with the formation of a theoretical framework, then to design a work plan that we had for weeks and with this work plan, to define the activities."—Alexandra

In category B, students brought together (consolidated) knowledge to give it to others. While, in category C, they applied it, that is, taking the learned theory into practice. Developing the project, they planned to solve community health problems.

"I think that at this point where I had this subject, for me it was very important because I managed to compact all the knowledge that I already had."—Alexandra

"The truth is doing… it is practice. One needs bases, and those bases as they were provided to us in an interactive way, it seems to me that it was the best way, trying to make us understand things from a previous experience. But now you really learn, and you realize things when you are in practice, when you must go, you have to start writing, you have to start looking for ways to solve those problems."—Camilo

In categories D and E, the students integrated knowledge, values, and previous personal and professional experiences. This integration was enhanced when building close relationships with community members during the rotation. Particularly, in category E there was a constant process of guided self-reflection, and feedback in multiple ways (student-community, tutor-community, between students and tutor–student).

"Beyond focusing solely on the patient and their disease, it is the ability to integrate all of the academic and scientific background with my previous experiences as a human being, not only in the university, in school and in the family, but in a comprehensive integration of past and newly achieved experience, thanks to a better interrelation with people in their communities."—Carlos

"At the end, when it was over, we also talked with the tutor about it, how I felt during the activity, the problems we had seen, and everything that happened that day...That was fundamental."—Alexandra

### Skills

In category A, students mainly developed professional skills: scientific writing and medical practical skills. In category B, students also strengthened professional skills in relation to patient education, and social communication skills.

"Another day, to remove some stitches from a man. These are things that we also learned to loosen up in medical skills."—Lina

"I realized that I knew many treatments, but I did not know how they were applied... So, it is really sitting down to educate someone, for someone to understand you, to say it in terms that persons in the community understand.”—Silvia

In categories C and D, other social skills were strengthened. In category C, empathy with service orientation and in category D, leadership, and conflict management. Sharing leadership, identified as a challenge, also stood out as a key skill.

"I had never considered it, but I took the role of leader with my group, and I was the one that tried to lead everyone towards some goal... Although I assumed the role of leader, others also assumed leadership in aspects where they had more knowledge. For example, a project where we had some stamps on the floor, none of us had any idea, except one who had the contacts, knew what could be done, what was needed and what was not. Then, my colleagues also took on the role of leaders in different moments."—Andrés

In category E, the student also developed social skills of empathy with the rest of the healthcare team, to provide comprehensive and humanized care. Besides, personal skills such as self-awareness and self-confidence were developed.

"In the end, I realized that I can be a leader for them. Tomorrow I will be able to manage a group, which is what I am going to do in my rural area."—Alexandra

## Outcome space

The students’ various ways of experiencing learning, along with their structural relationships, are depicted within an *outcome space* comprising five distinct *categories of description*. Category E ranked as the most complex, while category A ranked as the least complex. The learning experiences of higher levels included those of lower levels, but the same did not happen the other way around, the lower ones did not include those of higher levels, therefore, they were less inclusive.

Furthermore, this research distinguished between transformative learning (category E) and non-transformative learning (categories A, B, C, and D).

In category A, Fulfilling the Requirements—"Learner", the challenges were of less intensity and duration. The learning strategies and the relationships that were established were only a function of the fulfillment of the tasks. Developing technical skills predominated.

In category B, Educating the Community—"Teacher", the challenges were mainly related to the role assumed. After consolidating learned knowledge, the student made adaptations in a short time to overcome challenges. Relationships were collaborative and did not require synergies or deep ties, and skills developed concentrated in the areas of health education and communication.

In category C, Solving Health Problems—"Hero", the students generally faced challenges of low intensity and duration. To overcome them, the students applied learned knowledge, which developed empathy and led to collaborative relationships. However, there was no reflection, and no emotional ties were established.

In category D, Joint Construction—“Builder”, the challenges were of high intensity and duration, leading the student to integrate knowledge, experiences, attitudes, and values to face them. This generated collaborative and synergistic relationships. However, the students did not recognize reflection processes.

Finally, in the deeper learning experience, category E: Personal Transformation—“Pilot”, the challenges were of greater intensity and duration. Requiring reflection, feedback, and the establishment of affective bonds to face them. This developed professional, personal, and social skills to make long-term adaptations, associated with personal transformations and complex meanings of learning.

## Discussion

The aim of the present study was to understand how medical students from a Colombian University experienced learning in a community health course in a service-learning setting. The results were grouped into five qualitatively different ways of experiencing learning (*categories of description*): Fulfilling the requirements- "Learner", Educating the community- "Teacher", Solving health problems- "Hero", Joint construction- "Builder", and Personal transformation- "Pilot."

Moreover, four *dimensions of variation* were identified as critical pedagogical aspects that develop more complex learning in the service-learning strategy: challenges faced by students, students’ learning strategies, relationships established with their peers, community, and tutor’s roles, and skills developed by students during the course.

The *dimensions of variation* highlighted the qualitatively different ways of experiencing learning. They also facilitated the establishment of relations between the five resulting *categories of description,* accounting for the complexity in the constructs, ranging from a higher level of greater complexity and inclusion to a lower and less inclusive level. The highest level corresponded to a transformative learning experience.

Transformation understood as a deep and structural change in the assumptions that permeate the thoughts, feelings, and actions of the individual (Mezirow, 1991). For this, three elements of the transformational process proposed by Kiely (2005) were considered: (1) dissonances of high intensity and duration, “an activating event that typically exposes a discrepancy between what a person has always assumed to be true and what has just been experienced, heard or read” (Cranton, 2002, p. 66); (2) reflection, defined as the exploration of problems in a deep and critical way and examining alternative forms of thought, feelings, and actions, not previously considered (Naudé, 2015), and (3) the emotional process, as a result of relationships and bonding (García-Romero & Lalueza, 2019).

The findings indicate that a student’s transformation occurs when exposed to high-intensity, prolonged challenges, which prompt the use of complex learning strategies, including reflection, the formation of strong interpersonal relationships, and the development of a diverse set of skills. This process highlights the path to achieving various levels of complexity in community health learning through service-learning experiences.

Initially, a student may experience learning in a specific way, but as they face more intense challenges requiring increasingly complex learning strategies and reflection, they develop a broader range of skills. Throughout this process, they form strong relationships with others involved in their learning journey, leading to varied and more complex learning experiences, ultimately culminating in transformation.

This individual progression underscores a key finding of our study: students experience community health learning in a service-learning strategy in more than one way, and their individual experiences evolve over time. When a student discerns more critical aspects of learning and its features, they increase the likelihood of engaging with learning in a more developed manner, potentially reaching transformational learning. These findings contribute additional insights to previous studies that identified transformation processes in other service-learning contexts (Kiely, 2005; Naudé, 2015; Trigos-Carrillo et al., 2020) and provide information on how to facilitate such changes.

Several authors argue that training health professional students is more effective when developed within the communities where the professionals will go back to live and practice (Ellaway et al., 2013; Lalueza et al., 2016). In this way, TSL environments for health professionals’ education offer potential venues to bridge the gap between community health needs, educational demands, and the provision of health services.

In this study, results suggested that the TSL strategy allowed some students to develop not only academic and professional skills but also to acquire personal and social skills, which made them more self-aware, community-engaged, and ultimately socially accountable. The development of similar skills was reported in numerous studies that contemplate educational approaches aiming to connect students with the community (Ahmad et al., 2018; Arja et al., 2018; Carlisle et al., 2017; Chiva-Bartoll et al., 2019; Reinoso & Fonseca, 2019; Trigos-Carrillo et al., 2020).

As discussed, students reported that practical learning within a different sociocultural reality disrupts preconceptions and required them to seek new ways to deal with problems. Even more, some students developed professional identities corresponding to the type of doctor they aspired to become (teamwork, engagement in health education, and leadership in rural health teams), reflecting on the comprehensiveness and humanization of care when providing services to individuals. This is consistent with a previous study, where medical students developed a professional identity that was reflected in the values, behaviors with patients and in the change of roles they assumed in the community (Greenhill et al., 2018).

Other service-learning courses seeking community intervention skills development have found that the key pedagogical element for meaningful transformational learning, is critical reflection processes supported by meaningful interpersonal relationships with the tutor, academic peers, and community members (Reinoso & Fonseca, 2019; Trigos-Carrillo et al., 2020). Critical reflection and trust-based relationships between these actors has also been highlighted by community stakeholders as a determinant for longstanding partnerships between academia and communities working together towards wellbeing (Reinoso -Chávez et al., 2023). For this, continuous feedback from faculty, students, and community is required. This is consistent with elements of the transformational process (Kiely, 2005). Relationships play a fundamental role that in personal transformation (Cranton, 2002); therefore, it is important to highlight that learning within the community setting with the tutor as a role model makes a significant difference in learning outcomes (Reinoso & Fonseca, 2019; Trigos-Carrillo et al., 2020).

Despite the potential for personal transformation in the learning experience, not all students achieved it. Although transformative learning is not the only objective in adult education, it is one of the primary goals (Cranton, 2002) and it is a desirable result in the training of health professionals. Consequently, an intentional shift in the curricula of health sciences programs is necessary to create or increase opportunities for students to be engaged in their transformation (Van Schalkwyk et al., 2019). We have insisted that teachers play a pivotal role in students’ learning processes (Held et al., 2019). Therefore, a cultural transformation in educational leadership is required, to be open for dialogue, reflection, and experimentation while promoting the personal and professional growth of individuals (Polyzoi & Magro, 2015). In other words, teachers, supported by their institutions and the society, must create safe environments where participants may adopt and integrate into their practices the experienced changes (Cranton, 2002; Frenk et al., 2010).

The evolving needs of health professions education must keep pace with the changing health needs of the societies served by these professionals. Engaging all stakeholders—students, educators, patients, and families—is crucial (Nadarajan et al., 2024). To drive meaningful curriculum improvements, it is essential to understand students’ learning experiences in real-world scenarios (Nauhria et al., 2021; Pham et al., 2023). Such insights can facilitate ongoing curriculum review and, when necessary, program reforms, ensuring that educational programs remain responsive and effective. Ultimately, this approach helps in developing a well-trained healthcare workforce that can meet emerging health needs (Munangatire & McInerney, 2021).

## Conclusions: Implications for healthcare professions education

The findings of this study have implications for educational institutions that offer health sciences programs, particularly in the field of medicine, and for those responsible for curricula development since they are directly responsible for creating teaching–learning opportunities in similar contexts as the described here.

Learning community health in service-learning environments can be experienced in multiple ways, some of which are more complex than others. One of the main goals of education is to help students experience learning in the most powerful meaningful way (Orgill, 2012), and this course has proven to develop highly complex professional learnings, as well as personal and social competencies such us technical medical competencies, aspects of leadership, self-esteem, relationship building, among other things.

Therefore, looking forward transformational learning experiences, the described pedagogical conditions must be taken in consideration in curricula design to maximize students’ opportunities to experience the pedagogical critical aspects explored such as high-intensity dissonances, variation in learning and teaching strategies, opportunities to develop meaningful relationships and broaden skillset.

Furthermore, this requires institutional support for teachers to focus on the preparation of the experiential learning strategies that develop student`s desired capabilities. Likewise, tutors need training in reflective strategies, constructive feedback practices, and in capabilities to respond to students’ cognitive and emotional outcomes during the learning experience.

This study presents further understanding on how undergraduate medical students experience health community learning in a service-learning environment and the educationally critical aspects to increase probability of transformational processes in all those involved. Understanding students’ learning experiences from their own perspective is central to effective curricula improvements.

## Limitations

This research was conducted on undergraduate medical students from a single academic context. Future studies could expand the scope to experiences of students from various undergraduate medical schools’ programs with service-learning approaches and including the perspectives of communities’ stakeholders and faculty members. Further research in situated teaching and learning processes may continue to guide required improvements in medical education and thus respond to the current learning demands for health professionals that may better serve the changing health needs of communities.

## Positionality and reflexivity statement

Claudia Liliana Jaimes Peñuela, MD, M.H.P.E is a family physician and assistant professor of the Departments of Family Medicine and Public Health and Medical Education in the School of Medicine at the Universidad de La Sabana, Colombia. She has been a facilitator of community learning experiences for undergraduate and graduate medical students for 7 years, in diverse populations with different degrees of vulnerability and was recently named lead professor to coordinate community medical practice in the current school of Medicine curriculum.

Francisco Lamus Lemus, MD, M.P.H, M.H.P.E is a pediatrician and associate professor of the Department of Family Medicine and Public Health in the School of Medicine at the Universidad de La Sabana, Colombia. Researcher with a seasoned background in medical education, public health, maternal and child health, and community engagement spanning 25 years in leading community-based primary health projects. He has participated in the design, development, and evaluation of service-learning experiences for undergraduate and graduate health sciences students in similar contexts since the beginning of the community health program of the university’s medical school.

Natalia Reinoso Chávez is a community psychologist, lecturer and researcher on Qualitative Research, Health and Culture, and Community Psychology. She has been supporting participatory community processes for more than 10 years and a lecturer for 8 years. She has co-designed and studied service-learning strategies in partnership between academia and community members.

## Supplementary Information

Below is the link to the electronic supplementary material.

Supplementary file1 (DOCX 19 KB)

## Data Availability

The data supporting the findings of this study are available from the first author upon request.
