# Supplementary material for: Community health learning experiences of Colombian undergraduate medical students. A phenomenographic research study
Source: Adv Health Sci Educ Theory Pract. 2025 Feb 14;30(5):1473–95. doi: 10.1007/s10459-024-10395-3 (PMC12572061; doi:10.1007/s10459-024-10395-3)
Supplement: Supplementary file 1 — Supplementary file1 (DOCX 19 KB) [file 10459_2024_10395_MOESM1_ESM.docx]

**Running head (shortened title):** Experiences of community health learning

**Title:** Community health learning experiences of Colombian undergraduate medical students. A Phenomenographic research study.

**Journal name:** Advances in Health Sciences Education. Theory and Practice.

**Authors:** Claudia Liliana Jaimes-Peñuela^1^, Francisco Lamus-Lemus^1^ & Natalia Reinoso-Chávez^2^

**Affiliations:** ^1^ Universidad de La Sabana, Department of Family Medicine and Public Health, School of Medicine, Chía, Cundinamarca, Colombia; ^2^ Independent researcher and scholar

**Correspondence:** Universidad de La Sabana, Chía, Cundinamarca, Colombia; +57 601 8615555; [francisco.lamus@unisabana.edu.co](mailto:francisco.lamus@unisabana.edu.co)

**Appendix 1**

Interview guide exploring community health learning experiences

| **Opening questions**   - Why did you choose the community you worked with? - Describe your community health learning experience. - What was it like working with that community? - How would you describe your personal experience of community health learning? - What was your aim for community health learning?   **Follow-up questions**   - Can you describe a specific situation in your community health learning experience that was particularly challenging or rewarding? - What indicators or evidence do you use to determine that you have learned something? - What learning methods do you use and why in relation to community health learning? - Have you made any changes to your learning approaches? Why or why not? - What or who does your community health learning experience impact on? - How confident do you feel about community involvement in your learning, and why? - In what ways has your experience in community health learning influenced your approach to future projects or career plans? |
| --- |
